# Ail and PagC-Related Proteins in the Entomopathogenic Bacteria of *Photorhabdus* Genus

**DOI:** 10.1371/journal.pone.0110060

**Published:** 2014-10-15

**Authors:** Annabelle Mouammine, Anne Lanois, Sylvie Pagès, Bénédicte Lafay, Virginie Molle, Marc Canova, Pierre-Alain Girard, Bernard Duvic, Alain Givaudan, Sophie Gaudriault

**Affiliations:** 1 INRA, UMR Diversité, Génomes et Interactions Microorganismes-Insectes (DGIMI), Montpellier, France; 2 Université Montpellier 2, UMR Diversité, Génomes et Interactions Microorganismes-Insectes (DGIMI), Montpellier, France; 3 Université de Lyon, Écully, France; 4 CNRS, UMR5005 - Laboratoire Ampère, École Centrale de Lyon, Écully, France; 5 Laboratoire de Dynamique des Interactions Membranaires Normales et Pathologiques, Universités de Montpellier 2 et 1, CNRS, UMR 5235, Montpellier, France; Cornell University, United States of America

## Abstract

Among pathogenic *Enterobacteriaceae*, the proteins of the Ail/OmpX/PagC family form a steadily growing family of outer membrane proteins with diverse biological properties, potentially involved in virulence such as human serum resistance, adhesion and entry into eukaryotic culture cells. We studied the proteins Ail/OmpX/PagC in the bacterial *Photorhabdus* genus. The *Photorhabdus* bacteria form symbiotic complexes with nematodes of *Heterorhabditis* species, associations which are pathogenic to insect larvae. Our phylogenetic analysis indicated that in *Photorhabdus asymbiotica* and *Photorhabdus luminescens* only Ail and PagC proteins are encoded. The genomic analysis revealed that the *Photorhabdus ail* and *pagC* genes were present in a unique copy, except two *ail* paralogs from *P. luminescens*. These genes, referred to as *ail1*_Pl_ and *ail2*_Pl_, probably resulted from a recent tandem duplication. Surprisingly, only *ail1*_Pl_ expression was directly controlled by PhoPQ and low external Mg^2+^ conditions. In *P. luminescens*, the magnesium-sensing two-component regulatory system PhoPQ regulates the outer membrane barrier and is required for pathogenicity against insects. In order to characterize Ail functions in *Photorhabdus*, we showed that only *ail2*_Pl_ and *pagC*_Pl_ had the ability, when expressed into *Escherichia coli*, to confer resistance to complement in human serum. However no effect in resistance to antimicrobial peptides was found. Thus, the role of Ail and PagC proteins in *Photorhabdus* life cycle is discussed.

## Introduction

Various surface-exposed components present in the outer leaflet of the outer membrane play a crucial role in Gram-negative bacteria survival. Surface components have a dual role in virulent bacteria, first as factors maintaining the proper architecture of the outer membrane and as virulence factors [1]. About 50% of the outer membrane mass of Gram-negative bacteria consists of proteins, either lipoproteins that are anchoring the outer membrane to the underlying peptidoglycan or, integral membrane proteins [2]. The hallmark of integral outer membrane proteins is the folding into anti-parallel β-barrels [2], [3]. The most abundant integral membrane proteins of the bacterial outer membrane are porins, which are essentially trimeric β-barrels (16 or 18 β-strands) forming channels with various grades of selectivity [3]. Other barrel proteins having monomeric structure and fewer strands have been investigated, most displaying a specific function not related to the diffusion of hydrophilic molecules [3].

The family of related outer membrane proteins Ail/OmpX/PagC belongs to the latter category and was initially described in organisms like *Yersinia enterocolitica* and *Yersinia pseudotuberculosis* (Ail), *Salmonella* Typhimurium (Rck, PagC), *Escherichia coli* (OmpX, Lom) and *Enterobacter* (OmpX) [4]. These proteins display small size (from 15 to 18 kDa) and fold in eight β-barrels. Moreover, the Ail/OmpX/PagC proteins appear to be important for virulence by neutralizing host defense mechanisms. Ail from *Y. enterocolitica* promotes adhesion to and entry into eukaryotic tissue culture cells [5], [6]. PagC from *S.* Typhimurium is responsible for survival in macrophages [7], [8]. OmpX from *Enterobacter aerogenes* induces a β-lactam resistance mediated by a decrease in the porin production [9]. Lom from bacteriophage λ participates in *E. coli* adhesion to human buccal epithelial cells [10]. However, clear separation of functions between the different members of the Ail/OmpX/PagC family is not obvious. Indeed, Ail from *Y. enterocolitica* [5], [6], [11] and from *Yersinia pestis* [12], Rck from *S.* Typhimurium [6], PagC from *S. enterica* serovar Cholerasuis [13] are responsible for conferring resistance to complemented-mediated killing, but this property is not shared by PagC from *S.* Typhimurium, OmpX from *E. cloacae* or Lom from *E. coli* [6]. This discrepancy is probably due to gene annotation, which does not rely on an exhaustive phylogenetic analysis.

The expression of genes encoding outer membrane proteins has been found to be under a complex transcriptional regulation, acting as an adaptive response toward environmental physical attack of cell integrity. Modulation of expression of abundant outer membrane proteins such as porins are generally transcriptionally regulated by the two-component regulatory system OmpR-EnvZ and small RNAs [14]. Regulatory pathways controlling the expression of genes encoding proteins from the Ail/OmpX/PagC family have been elucidated in some Gram-negative bacteria, but there is no convergence towards a common pathway. For instance, in *Y. enterocolitica* 8081, *ail* is regulated by temperature [15]. By contrast, *ail* from *Y. enterocolitica* O: 9 is not subjected to thermoregulation but is under the control of the OmpR transcription factor [16]. *ompX* is regulated by a small RNA in *S.* Typhimurium [17]. The regulation of *pagC* is under the control of the two-component system PhoP-PhoQ through SlyA in *S.* Typhimurium [18]. This PhoPQ system has been extensively studied in bacteria and especially in *S.* Typhimurium, in which the response regulator PhoP regulates about 3% of *Salmonella* genes, subdivided into the PhoP-activated genes, *pag*, and the PhoP-repressing genes, *prg* [19]. In a variety of Gram-negative bacterial pathogens, numerous PhoP-regulated genes encode enzymes involved in LPS modifications [20]–[23], as well as several secreted and outer membrane proteins [24].

*Photorhabdus* (*Enterobacteriaceae*) is an insect pathogen living in a symbiotic association with entomopathogenic nematodes *Heterorhabditis* [25]. *Heterorhabditis bacteriophora* nematodes invade insect larvae and regurgitate bacteria from their gut directly into the hemolymph, the insect blood [26]. The bacteria overcome the insect immune system and colonize the insect body cavity leading to lethal septicemia [25]. Bacterial virulence factors and insecticidal toxins also participate to the insect death [27], [28]. Once the insect host is dead, bacteria bioconvert the tissues and the nematode partner feeds off the bacteria while nematode reproduction occurs through several generations [29]. *Photorhabdus* also successfully competes with saprophytic scavenging organisms. It produces antimicrobial factors in order to kill any invading and competing microbes [25]. Several rounds of nematode reproduction and bacterial replication lead to a new generation of infective juvenile (IJ) nematodes. *Photorhabdus* bacteria colonize specifically the posterior-intestinal cells of the maternal adult nematode before re-associating with the new IJ [30], [31]. The dual requirement for symbiosis and virulence makes *Photorhabdus* an excellent model organism for studying host-bacteria interactions. The genus *Photorhabdus* comprises four distinct species: *Photorhabdus temperata, Photorhabdus luminescens*, *Photorhabdus heterorhabditis* and *Photorhabdus asymbiotica* [32]. Although all four are highly pathogenic to insects, *P. asymbiotica* also causes infection in humans [33]–[35].

In an attempt to identify host-interacting bacterial proteins, we were interested in proteins from the Ail/OmpX/PagC family of *Photorhabdus* genus. Duchaud *et al.* [36] already described three Ail-like homologs in *P. luminescens* strain TT01. Thus, we exhaustively searched for proteins from the Ail/OmpX/PagC family encoded in the genomes of *P. luminescens* strain TT01 [36] and *P. asymbiotica* strain ATCC43949 [37]. Analysis of Ail/OmpX/PagC phylogeny supports a robust annotation showing that the *Photorhabdus* genus only encodes Ail and PagC orthologs. Then, we present the first detailed investigation into the role and the regulation of Ail and PagC proteins from *Photorhabdus.*

## Materials and Methods

### Bacterial strains, plasmids, primers and growth conditions

The strains and plasmids used in this study are listed in Table 1. All primers used in this study (Eurogentec) are listed in Supplemental Table 1. *Photorhabdus* and *Escherichia coli* strains were routinely grown at 28°C and 37°C, respectively, in Luria-Bertani (LB) broth or on LB solid medium. *Photorhabdus* was grown in M9 liquid medium with concentrations of MgSO_4_ (10 µM and 10 mM) and supplemented with 0.1% casamino acids, 0.41 mM nicotinic acid, 9.1 mM sodium pyruvate, 0.2% glycerol and 0.1 mM CaCl_2_. When required, antibiotics were used at the following final concentrations: kanamycin (Km) 20 µg/ml, gentamicin (Gm) 30 µg/ml, ampicillin (Ap) 100 µg/ml.

**Table 1:** Strains and plasmids used in this study.

Strain or plasmid	Genotype or relevant characteristics	Source or reference
***P. luminescens*** ** strain**		
TT01	Strain isolated from the nematode *Heterorhabditis bacteriophora* THO1 in Trinidad; wild-type form	[75]
*phoP*	TT01 *phoP*::cat, *phoP* mutant	[21]
TT01/P*_ail1_*_Pl_ -*gfp*[AAV]	Conjugative strain, TT01 carrying P*_ail1_*_Pl_ -*gfp*[AAV] plasmid, Km^R^	this work
TT01/P*_ail2_*_Pl_ -*gfp*[AAV]	Conjugative strain, TT01 carrying P*_ail2_*_Pl_ -*gfp*[AAV] plasmid, Km^R^	this work
TT01/P*_lac_* -*gfp*[AAV]	Conjugative strain, TT01 carrying P*_lac_* -*gfp*[AAV] plasmid, Km^R^	[51]
*phoP*/P*_ail1_*_Pl_ -*gfp*[AAV]	Conjugative strain, *phoP* carrying P*_ail1_*_Pl_ -*gfp*[AAV] plasmid, Km^R^	this work
*phoP*/P*_ail2_*_Pl_ -*gfp*[AAV]	Conjugative strain, *phoP* carrying P*_ail2_*_Pl_ -*gfp*[AAV] plasmid, Km^R^	this work
***E.coli*** ** strain**		
XL1Blue	F′ *proAB lacI* ^q^ *Z*ΔM15 Tn*10*(Tet^R^)	Laboratory stock
BL21 (DE3) pLysS	F^−^ *dcm ompT hsdS*(r_B_^−^m_B_^−^) *gal* λ(DE3) (pLysS Cam^R^)	Laboratory stock
WM3064	*thrB*1004 *pro thi rpsL hsdS lacZ*ΔM15 RP4-1360 Δ(*araBAD*)567 Δ*dapA*1341::[erm pir (wt)]	[76]
**Plasmid**		
pUC19	High copy number vector, Ap^R^	Laboratory stock
pUC-*ail*_Yp_	0.65 kb PCR fragment obtained with y1324-*Pst*I and y1324-*Sac*I primers and inserted between the *Pst*I and *Sac*I sites of pUC19	this work
pUC-*ail1*_Pl_	0.7 kb PCR fragment obtained with plu2480-*Pst*I and plu2480-*Sac*I primers and inserted between the *Pst*I and *Sac*I sites of pUC19	this work
pUC-*ail2*_Pl_	0.7 kb PCR fragment obtained with plu2481-*Pst*I and plu2481-*Sac*I primers and inserted between the *Pst*I and *Sac*I sites of pUC19	this work
pUC-*pagC*_Pl_	0.8 kb PCR fragment obtained with plu1967-*Pst*I and plu1967-*Sac*I primers and inserted between the *Pst*I and *Sac*I sites of pUC19	this work
pUC-*ail*_Pa_	0.65 kb PCR fragment obtained with PAU_02047-*Pst*I and PAU_02047-*Sac*I primers and inserted between the *Pst*I and *Sac*I sites of pUC19	this work
pUC-*pagC*_Pa_	0.7 kb PCR fragment obtained with PAU_02601-*Pst*I and PAU_02601-*Sac*I primers and inserted between the *Pst*I and *Sac*I sites of pUC19	this work
pPROBE-*gfp*[AAV]	Plasmid (pBBR1 replicon) containing *gfp*[AAV] gene downstream from a multiple cloning site, Km^R^	[77]
P*_lac_*-*gfp*[AAV]	pPROBE with *gfp*[AAV] under the control of P*_lac_* promoter; Km^R^	[51]
P*_ail1_*_Pl_ -*gfp*[AAV]	pPROBE with *gfp*[AAV] under the control of *ail1* (plu2480) gene promoter; Km^R^	this work
P*_ail2_*_Pl_ -*gfp*[AAV]	pPROBE with *gfp*[AAV] under the control of *ail2* (plu2481) gene promoter; Km^R^	this work
pETPhos	pET28 replicon, Ap^R^	[48]
P_T7_PhoP-His	pET28 producing PhoP(His-tag) in N-terminal under the control of T7 promoter; Ap^R^	this work

### Inference of the evolutionary relationships of Ail, PagC and OmpX-related proteins

Ail, PagC or OmpX annotated proteins in *Photorhabdus luminescens* TT01 (plu1967, plu2480, plu2481) and *Photorhabdus asymbiotica* ATCC43949 (PAU_02047 and PAU_02601) were identified and retrieved using the protein family analysis tool PipeAlign [38]. Outputs were pooled and resulting dataset were curated for protein multiple occurrences. The sequences were aligned using ClustalW [38] followed by manual curation. The sequence alignment was generated by Gblocks [39] and unambiguously aligned amino acid sites were retained for phylogeny inference using the maximum likelihood method implemented in PhyML [40]. Analyses were engendred under the LG model of amino acid replacement [41] with a gamma distribution of evolutionary rates across sites [42]. Internal branch supports were evaluated using the approximate Likelihood Ratio Test [43].

### Molecular techniques and RNA preparation

DNA manipulations were carried out as previously described [44]. Plasmids were introduced into *E. coli* WM3064 (Table 1) by transformation and transferred to *P. luminescens* TT01 by filter mating [45]. All constructs were sequenced by Eurofins MWG Operon (Ebersberg, Germany). Total RNA was extracted with TRIzol reagent according to manufacturer's instructions (Invitrogen) and purified using RNeasy miniprep kit (Qiagen), including a DNase I treatment step. For each RNA preparation, we assessed DNA contamination by carrying out a control PCR. The quantity and quality of RNA were assessed with a NanoDrop 2000 spectrophotometer (Thermo Scientific) and an Agilent 2100 Bioanalyzer with the RNA 6000 Nano LabChip kit (Agilent), respectively. Material for real-time quantitative polymerase chain reaction (RT-qPCR) analysis was prepared by extracting total RNA from the *P. luminescens* wild-type strain and the *phoP* mutant grown in Luria broth (OD_540_ = 0.5–0.8). The gene expression level was evaluated during the growth phase.

### Real-time quantitative polymerase chain reaction analysis

RT-qPCR was performed in two steps. First, the cDNA was synthesized from 500 ng of total RNA, with Super Script II Reverse Transcriptase (Invitrogen) and random hexamers (100 ng/µl) (Applied Biosystems). We then carried out qPCR in triplicate with the LightCycler 480 SYBR Green I Master kit from Roche Diagnostics, with 1 µl of cDNA synthesis mixture (diluted 1∶100) and 1 µM of specific primers for the genes studied (Supplemental Table 1). The enzyme was activated by heating for 10 min at 95°C. All qPCRs were performed in three technical replicates, with 45 cycles of 95°C for 5 seconds, 60°C for 5 seconds and 72°C for 10 seconds, and were monitored with the Light Cycler 480 system (Roche). Melting curves were analyzed for each reaction and each curve contained a single peak. The data for each sample were expressed relatively to the expression level of *gyr,* using REST software 2009 [46] as previously described [47]. This method provided a relative quantification of the target gene expression with respect to a reference gene (*gyrB*).

### Overexpression and purification of PhoP recombinant protein

The entire coding region of *phoP* gene from TT01 strain was amplified by PCR and digested by *Nde*I and *Bam*HI. The obtained PCR product was ligated into the same site of the expression vector, pETPhos [48] inserting a His-tag in the N-terminal part of PhoP thus generating the plasmid P_T7_PhoP-His. The recombinant plasmid encoding a PhoP-His fusion protein was transformed into *E. coli* BL21 (DE3) pLysS cells. At an OD between 0.5–0.8, the expression of PhoP-His was induced by adding Isopropyl-beta-D-thiogalactoside at 0.5 mM. An overnight induction was then performed at 18°C. Bacterial culture was centrifuged at 7,000×g for 15 min at 4°C and washed twice in resuspension buffer (Tris 5 mM pH 7.5, NaCl 300 mM, glycerol 10%, Imidazole 10 mM). The pellet was frozen at −80°C for 30 min, then suspended in 5 ml resuspension buffer and lysed by sonication during 10 min at 4°C. Lysis products were centrifuged at 10,000×*g* during 30 min at 4°C. Five hundred µl of pre-equilibrated beads of Ni-NTA agarose (Qiagen) in wash buffer (Tris 5 mM pH 7.5, NaCl 300 mM, glycerol 10%, Imidazole 15 mM) were added to the supernatant fraction and incubated during 45 min with shaking at 4°C. The fraction was centrifuged at 500×*g* during 2 min at 4°C and wash 5 times with wash buffer. Protein was eluted twice in 1 ml elution buffer (Tris 5 mM pH 7.5, NaCl 300 mM, glycerol 10%, Imidazole 200 mM). Concentration of recombinant protein was assessed by Bradford assay and controlled by SDS-PAGE gel. Recombinant proteins were conserved at −80°C until use.

### Electrophoretic mobility-shift assays

The promoter of *ail1*_Pl_ was PCR-amplified from the genomic DNA of TT01 strain using specific primers (Supplemental Table 1) and purified using the High Pure PCR Product Purification kit (ROCHE). The 5′ ends of DNA fragment were labeled using [γ-^32^P] ATP and T4 polynucleotide kinase (Promega). Radioactive DNA probe (2000 cpm/ml), 200 ng of poly(dI-dC)-poly(dI-dC) (SIGMA) and different amounts of PhoP-His were mixed with binding buffer (50 mM tris-HCl pH 8, 50 mM KCl, 50 µg/ml BSA) in a total 20 µl volume and incubated for 20 min at room temperature. The mixture was then loaded onto a native 6% (w/v) polyacrylamide TBE precast Gel (Invitrogen) and electrophoresed in 1% TBE (Tris-Borate-EDTA) buffer for 1 h at 100 V. Radioactive species were detected by autoradiography. PhoP-His was activated by *in vitro* phosphorylation with acetyl phosphate as previously described [49].

### Construction of plasmids expressing *gfp*[AAV] under the control of *ail1*_Pl_ or *ail2*_Pl_ promoters

Plasmids expressing the reporter gene *gfp*[AAV] under the control of *ail1*_Pl_, *ail2*_Pl_ or *lac* gene promoters were constructed using a previously described method [50]. The construction of P*_lac_*-*gfp*[AAV] has been described elsewhere [51]. Briefly, DNA fragments located upstream from *ail1*_Pl_ (360 bp) *and ail2*_Pl_ (358 bp) were amplified by PCR from *P. luminescens* TT01 genomic DNA with primers containing the *Eco*RI and *Bam*HI restriction site (Supplemental Table 1). The PCR products were *Eco*RI- and *Bam*HI-hydrolyzed and inserted into the corresponding sites of pPROBE-*gfp*[AAV]. Finally, P*_lac_*-*gfp*[AAV], P*_ail1_*_Pl_-*gfp*[AAV], P*_ail2_*_Pl_-*gfp*[AAV] were transferred by filter bacterial mating [45] in *P. luminescens* TT01 wild type and *phoP* strains.

### Quantification of *ail1*_Pl_ and *ail2*_Pl_ expression in bacterial populations grown in different media

Wild-type strains carrying either P*_ail1_*_Pl_-*gfp*[AAV], P*_ail2_*_Pl_-*gfp*[AAV] or P*_lac_*-*gfp*[AAV] constructs were cultured in black-sided, clear-bottomed 96-well plates (Greiner). For each well, a 1∶20 dilution of an overnight culture was added to the M9 minimal medium supplemented with kanamycin and different concentrations of MgSO_4_ or to LB medium supplemented with kanamycin. Then, the plates were incubated at 28°C for 45 h with shaking on an orbital shaker, in an Infinite M200 microplate reader (Tecan). Absorbance at 600 nm and GFP fluorescence intensity (excitation at 485±4.5 nm; emission at 520±10 nm) were measured every 30 min. Specific fluorescence was obtained by dividing fluorescence units (at the maximum level of expression) by the absorbance value.

### Serum-killing assay

The serum-killing assay was performed as described previously [5] with overnight culture of *Escherichia coli* strain XL1Blue in LB with ampicillin 100 µg/ml at 37°C. For each bacterial strain, three independent assays were performed with human serum from human male AB sterile plasma (Sigma-Aldrich, reference: H4522) or with serum that was heat-treated to inactivate complement (56°C, 30 min). The number of viable bacteria after incubation with serum at 37°C for 60 min was calculated by serial dilution, plating on LB agar with ampicillin 100 µg/ml and counting the colony forming units (CFU). The degree of killing was calculated as follow: log kill  =  (log_10_ CFU per milliliter of initially added bacteria) - (log_10_ CFU per milliliter of bacteria surviving the incubation). The resistance was expressed as the difference in log kill between XL1-Blue harboring pUC19 incubated in 50% human serum and XL1Blue harboring the recombinant plasmid incubated either in 50% human serum or heat-inactivated serum.

### Cell association assays

The cell invasion and association assays were performed as described previously [52]. *Escherichia coli* strain XL1Blue were grown at 37°C in LB broth supplemented with ampicillin 100 µg/ml for 2.5 hours (10^8^ CFU/ml; optical density at 540 nm of 0.5; xponential-growth phase). Chinese hamster ovary cells (CHO) were grown and maintained at 37°C in RPMI Medium 1460-glutamax I (Gibco) complemented with 10% foetal bovine serum (Lonza), and 1% PenStrep 5000 U/ml (Gibco). *Spodoptera littoralis* cells derived from hemocytes (Sl2b) were grown and maintained at 28°C in G3 Medium pH 6,2 (TC100 2% from Gibco modified with 0.037% α-ketoglutaric acid, 0.04% β-fructose, 0.005% fumaric acid, 0.067% malic acid, 0.006% succinic acid, 0.26% sucrose, 0.02% choline chloride, 0.02% β-alanine, 0.035% sodium bicarbonate, 0.33% lactalbumin hydrolysate, and complemented with 5% foetal bovine serum, 0.016% penicillin, 0.006% streptomycin).

### Susceptibility to antimicrobial peptides

*In vitro* susceptibility to polymyxin B sulfate (Sigma), colistin methanesulfate (Sigma), cecropin A (Sigma), *Spodoptera frugiperda* cecropin B was evaluated by determination of minimal inhibition concentration as previously described [53].

## Results

### Phylogeny of Ail, PagC and OmpX-related proteins among Bacteria

**Figure 1:**
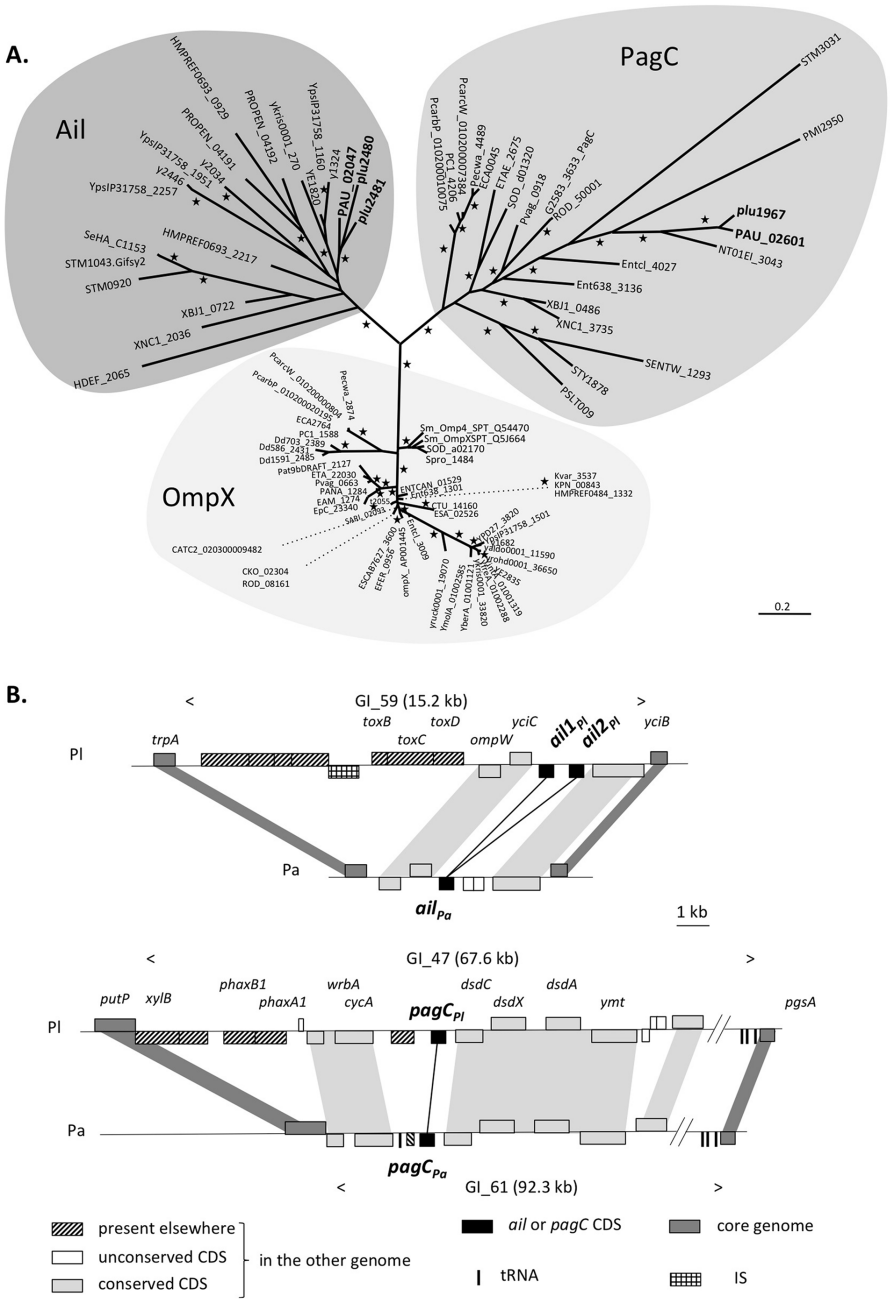
The *Photorhabdus* genus harbors *ail* and *pagC* genes. A. Evolutionary relationships of Ail, PagC and OmpX-related proteins. Stars indicate branch supports higher than 0.85 (used as significance threshold). The scale bar corresponds to the number of substitutions per amino acid residue site. B. Conserved genomic context of the *ail* (up) and *pagC* (bottom) genes in *Photorhabdus luminescens* TT01 (Pl) and *Photorhabdus asymbiotica* ATCC43949 (Pa) genomes. The boxes above and below the axis represent ORFs in the forward and reverse orientations, respectively. The names of some genes are indicated. The names of genomic islands (GI) previously described in the *P. luminescens* and *P. asymbiotica* genomes [55] are given.

Ail, PagC and OmpX homologs were searched in genome databases (see Material and Methods). Phylogenetic analysis (Fig. 1A) clearly separated the 89 proteins from 53 bacterial species into three well-supported groups. The Ail group recovered the y1324 canonical Ail protein described in *Y. pestis* KIM [4]. The PagC group contained the STM3031 PagC protein from *Salmonella* Typhimurum [54]. Finally, the OmpX group was characterized by several proteins annotated OmpX, such as Ent638_1301 from *Enterobacter* sp. 638. Interestingly, the protein y1682 of *Y. pestis* KIM usually named Ail clusters with the OmpX group [4]. Our phylogenetic analysis clearly indicates that all the *Photorhabdus* proteins clustered within the Ail group (plu2480 and plu2481 from *P. luminescens* and PAU_02047 from *P. asymbiotica*) and the PagC group (plu1967 from *P. luminescens* and PAU_02601 from *P. asymbiotica*).

### Genomic organization of *ail* and *pagC* genes in *Photorhabdus* genomes

Analysis of the *P. asymbiotica* ATCC43949 and *P. luminescens* TT01 genome sequences [36], [37] revealed that *ail* and *pagC* genes were located in conserved regions in the two *Photorhabdus* species, previously described as genomic islands in *P. luminescens* TT01 (Fig. 1B) [55]. Both insertion points and contents in the coding sequences were similar. The genomic islands from *P. luminescens* genomes were composed of additional genes encoding proteins potentially involved in infectious process. The *toxBCD* operon in the vicinity of the *ail* genes (GI_59) is involved in toxoflavin biosynthesis of *Burkholderia glumae* [56]. The *phaxA1B1* genes in the vicinity of the *pagC* (GI_47) encodes the XaxAB-like binary toxin with insecticidal and cytotoxic activity [57], [58].

The two *ail* genes from *P. luminescens*, referred to as *ail1_Pl_* and *ail2_Pl_* hereafter, shared 71% of nucleotidic identity and 66% of aminoacids identity and were separated by 517 nucleotides. This adjacent position (Fig. 1B) together with their closed clustering inside the Ail group (Fig. 1A) suggests a recent tandem duplication. By contrast, the *ail* gene from *P. asymbiotica* (*ail*_Pa_), the *pagC* genes of *P. luminescens* and *P. asymbiotica* (*pagC*_Pl_ and *pagC*_Pa_, respectively) were present in single copy.

### PhoP is directly regulating the expression of *ail1*_Pl_

**Figure 2:**
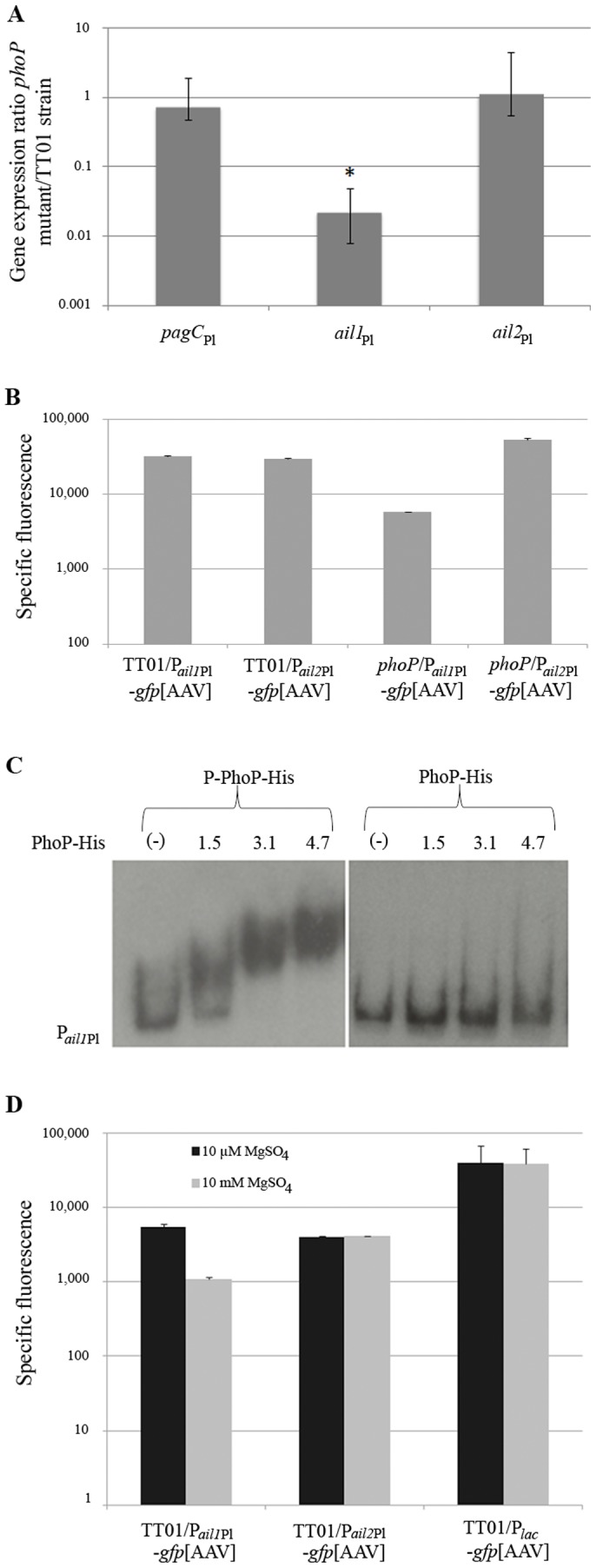
Only *ail1*_Pl_ is directly regulated by PhoP. **A**. RT-qPCR: *ail1*_Pl_ expression is PhoP-dependent. Total RNA from *phoP* mutant or TT01 wild-type strain of *Photorhabdus luminescens* was used for RT-qPCR analysis with internal primers specific for the indicated genes. mRNA levels were normalized against those of a reference gene (*gyrB*). Data are presented as a ratio of values for *phoP* mutant and TT01 wild-type strain. A ratio of 1 indicates no difference in expression level between both strains. The bars indicate standard errors calculated using Taylor's series. Significant differences (*p*-value <0.05) are indicated by asterisks (*). The relative quantification results were obtained from three independent experiments with the REST 2009 program. **B**. Gene transcription monitored by GFP quantification: *ail1*_Pl_ promoter region is positively regulated by PhoP. The dynamic expression of *ail1*_Pl_ and *ail2*_Pl_ promoter in TT01 and *phoP* backgrounds was monitored over time after growth in LB medium. Each histogram represents the specific fluorescence at the peak of expression for each condition. One experiment representative of more than three independent experiments is shown. Standard deviations represent technical replicates. **C**. PhoP-His binds the promoter region of *ail1*_Pl_. Electrophoretic mobility shift assay was carried out to test the binding of PhoP-His protein activated *in vitro* with ACP 10 mM (P-PhoP-His) or non activated PhoP-His (PhoP-His) on *ail1*_Pl_ promoter. The PhoP-His concentrations indicated are in micromolar. To ensure that the fixation is specific, we used BSA proteins and poly(dI-dC) in the binding buffer. **D**. *ail1*_Pl_ expression is higher at low MgSO_4_ concentrations. We evaluated the impact of low and high MgSO_4_ concentrations on *ail1*_Pl_ and *ail2*_Pl_ expression. Cultures diluted at 1/200 were grown in M9 minimal medium supplemented with 10 µM or 10 mM MgSO_4_. Each histogram represents specific fluorescence at the peak of expression for each condition. Experiments were realized at least three times.

Data on expression of genes encoding outer membrane proteins of the Ail/OmpX/PagC family remains limited except for PagC. Indeed, *pagC* gene in *S.* Typhimurium is a PhoP-activated gene [7]. A *phoP* mutant has been previously constructed and described for *P. luminescens* TT01 [21]. We therefore performed RT*-*qPCR to measure levels of *pagC*_Pl_, *ail1*_Pl_ and *ail2*_Pl_ mRNA and to calculate their ratio of values in *phoP* and wild type backgrounds of *P. luminescens* TT01 (Fig. 2A). Only the level of *ail1*_Pl_ transcript was lower in the *phoP* mutant than in the wild type strain, indicating that the *ail1*_Pl_ gene expression requires PhoP. Contrarily, *ail2*_Pl_ *and pagC*_Pl_ expression was not PhoP-dependent. The differential regulation of *ail* paralogs were independently confirmed by measuring expression of P*_ail1_*_Pl_-*gfp*[AAV] and P*_ail2_*_Pl_-*gfp*[AAV] fusions in wild-type and *phoP* backgrounds (Fig. 2B). Next, the possible direct binding of PhoP protein upstream of *ail1*_Pl_ was investigated. Electrophoretic mobility shift assays (EMSAs) were carried out to compare the interaction profiles of different amounts of PhoP protein on the 360-bp *ail1*_Pl_ promoter region (Fig. 2C). A recombinant N-terminal His-tag PhoP protein (PhoP-His) was first produced from P_T7_PhoP-His vector (Table 1). The PhoP-His protein was purified and phosphorylated *in vitro* by incubation with acetyl phosphate. Phosphorylation efficiency and dimer formation were assessed by migration on precast Phos-tag gel (Fig. S1). Then, different amounts of phosphorylated and unphosphorylated PhoP-His were mixed with radiolabeled *ail1*_Pl_ promoter. A gel shift pattern was observed when 1.5 µM of phosphorylated PhoP-His was added (Fig. 2C). No shifted bands were observed upon incubation with unphosphorylated PhoP-His. Therefore, PhoP-His protein can specifically bind to the promoter region of *ail1*_Pl_ and the active form of PhoP corresponds to the phosphorylated isoform.

### *ail1*_Pl_ expression is high at low MgSO_4_ concentrations

It has been shown that low concentrations of Mg^2+^ activate the expression of PhoP-dependent genes in *Salmonella* whereas high Mg^2+^ concentrations repress the system (for review see [59], [60]. In order to evaluate the role of Mg^2+^ concentration on *ail1*_Pl_ or *ail2*_Pl_ expression, wild-type strain containing P*_ail1_*_Pl_-*gfp*[AAV], P*_ail2_*_Pl_-*gfp*[AAV] or P*_lac_*-*gfp*[AAV] were grown in minimal medium M9 supplemented with 10 µM (activating concentration) or 10 mM (repressing concentration) of MgSO_4_ and gene expression was monitored by recording GFP fluorescence (Fig. 2D). We observed a 5-fold decrease of *ail1*_Pl_ expression at 10 mM MgSO_4_, whereas *ail2*_Pl_ expression was not dependent on the concentration of MgSO_4_. As observed for PhoP-activated genes in *S.* Typhimurium [19], low MgSO4 concentration increases *ail1*_Pl_ expression in *Photorhabdus*.

### *ail2*_Pl_ and *pagC*_Pl_ genes confer human serum resistance but no eukaryotic cell association phenotype

**Figure 3:**
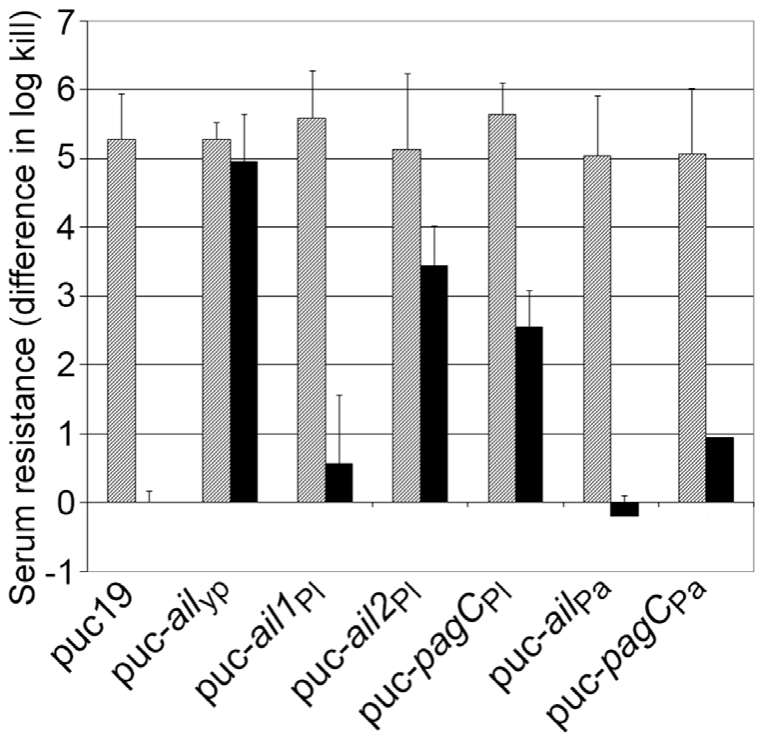
Human serum resistance of *Escherichia coli* XL1Blue strains carrying the plasmid pUC19 and its derivatives harboring *ail* or *pagC* genes. Overnight grown bacteria were tested for viability at 37°C in 50% serum (black histograms) or heat-inactivated serum (hatched histograms). The resistance was expressed as the difference in log kill between XL1-Blue harboring pUC19 incubated in 50% human serum and XL1Blue harboring the recombinant plasmid incubated either in 50% human serum or heat-inactivated serum. Means and standard errors of results from triplicate experiments are shown.

When introduced into *Escherichia coli*, individual members of the Ail/PagC/OmpX-related protein family have the property to confer resistance to human serum complement and to associate with eukaryotic cell [5], [6], [11], [13]. In order to test if the *ail* and *pagC* genes from *P. luminescens* and *P. asymbiotica* have similar phenotypes, we introduced *ail1*_Pl_, *ail2*_Pl_, *pagC*_Pl,_ *ail*_Pa_ and *pagC*_Pa_ under the control of the constitutive promoter P_lac_ into the high copy number pUC19 plasmid and transformed these pUC19 derivatives into an *E. coli* XL1blue strain. Active transcription of corresponding cloned genes in *E. coli* XL1Blue was controlled by real-time PCR (data not shown). We first evaluated the human serum resistance of the *E. coli* XL1Blue strains harboring the different derivative plasmids (Fig. 3). The positive control, *E. coli* XL1Blue expressing the *ail* gene from *Y. pestis* strain KIM, y1324 (*ail*_Yp_), showed an elevated serum resistance, similar to the one observed in all strains when the complement in the serum was heated-inactivated. Moreover, we observed intermediate serum resistance with the *ail2*_Pl_ and *pagC*_Pl_ genes whereas the resistance with the *ail1*_Pl_, *ail*_Pa_ and *pagC*_Pa_ genes was weak.

In order to assess if the association of *E. coli* XL1Blue to eukaryotic cells was affected by expression of *Photorhabdus ail* or *pagC* genes, association assays of mammals cells (CHO) and insect cells (Sl2b) were performed. *E. coli* XL1Blue expressing *ail*_Yp_ displayed association with CHO cells (% of association of 30.43±17.06) in regards to *E. coli* XL1Blue harboring the pUC19 plasmid (% of association of 0.57±0.05), which is consistent with previously published data [52]. None of the *Photorhabdus* over-expressed genes, *ail1*_Pl_, *ail2*_Pl_, *pagC*_Pl,_ *ail*_Pa_ and *pagC*_Pa_, was able to confer to *E. coli* XL1Blue strain an improved association with Sl2b cells to *E. coli* XL1Blue strain (average % of association of 10.60±6.80).

### *ail1*_Pl_, *ail2*_Pl_ and *pagC*_Pl_ do not confer antimicrobial peptide resistance

Since *P. luminescens* TT01 and *P. asymbiotica* ATCC43949 are pathogenic towards insect, the role of *ail* and *pagC* genes was studied by cultivating recombinant *E. coli* in insect hemolymph, which is analogous to the mammalian blood regarding the immune system. The growth of the *E. coli* XL1Blue derivatives was not affected when cultivated in sterile hemolymph of *Spodoptera littoralis* fifth-instar larvae (data not shown). Then, we compared the susceptibility to different antimicrobial peptides of the negative control *E. coli* XL1Blue/pUC19 and the *E. coli* XL1Blue harboring the different derivative plasmids. Similar minimal inhibitory concentrations were observed towards colistin (1.5–3 µg/ml), polymyxin B (0.1–0.2 µg/ml), cecropin A (6–12.5 µg/ml) or cecropin B from the Lepidoptera *Spodoptera frugiperda* (1.5–3 µg/ml).

## Discussion

### *Photorhabdus* genomes only harbor *ail* and *pagC* gene homologs

Our analysis of 89 proteins from 53 bacterial species belonging to the Ail/PagC/OmpX family clearly distinguished three sub-families. In the two whole-assembled genomes of *Photorhabdus*, both Ail and PagC proteins are encoded. Interestingly, in *P. luminescens* TT01, we identified two intra-genome homologs *ail1* and *ail2*. Such homologs can arise through duplication, where both gene copies are named paralogs, or by acquiring similar genes from outside sources through horizontal gene transfer, where both gene copies are named xenologs [61]. The adjacent position of *ail1*_Pl_ and *ail2*_Pl_ genes suggests recent tandem duplication. Duplication is an important hallmark of the genome plasticity of *P. luminescens* both in short-term adaptation and long-term evolution. Several dozen of duplicated genes have already been described in *P. luminescens* TT01 [36]. Interestingly, another gene encoding an outer membrane protein, the *ompF*-like gene, probably underwent a tandem duplication [62]. Characteristic features of an ancient whole-genome duplication were also detected in the *P. luminescens* TT01 genome [63]. In addition, a 275-kilobase single block duplication, with cryptic phenotypic consequences, was observed in phenotypic variants of *P. luminescens* [64].

### What are the factors regulating *ail/pagC* gene expression in *Photorhabdus*?

No data about transcription factors involved in the control of *ail*/*pagC* expression in the *Photorhabdus* genus are currently available. *P. asymbiotica* is considered an emerging human pathogen [34]. In an attempt to find host-interacting proteins that are relevant to either human or insect infections is interesting, Wilkinson *et al.* identified a thermoregulation of the secretion for its Ail-like homolog in *P. asymbiotica* ATCC43949 (Ail_Pa_ in our study) as Ail_Pa_ is secreted at 37°C but not at 30°C [37]. In our study, we decided to use the available *phoP* mutant of *P. luminescens* TT01 [21] to compare relative expression of *ail1*_Pl_, *ail2*_Pl_ and *pagC*_Pl_ in wild-type and *phoP* backgrounds. Only *P. luminescens ail1* expression is PhoP-dependent by contrast with *ail2*_Pl_ and *pagC*_Pl_. The *pagC* gene from *S.* Typhimurium is regulated by PhoP in a indirect way *via* SlyA [18]. Surprisingly, our electromobility shift assays showed that the His-tagged PhoP protein from *P. luminescens* directly binds to the promoter region of *ail1*. This is the first time that *ail* has been described to rely on the direct control of PhoP in Gram-negative bacteria. In addition, we monitored the kinetic of *ail1* expression in different culture conditions and determined that *ail1* expression was reduced in the presence of high concentrations of MgSO_4_. The environmental deprivation of Mg^2+^ as a signal activating the PhoP/PhoQ signal transduction cascade was first described in *Salmonella* [19]. In *Photorhabdus,* Derzelle *et al.* [21] showed that PhoP-dependent expression of the first gene of the *pbgPE* operon, involved in LPS modifications, relies on the Mg^2+^ concentrations in the culture medium. Like *pbgPE*, expression *ail1*_Pl_ is higher at low Mg^2+^ concentrations than at high concentrations. In *Salmonella* and *P. luminescens*, both the *phoP* gene and the *pbgPE* operon are involved in virulence in the mouse and the insect models, respectively [7], [21], [65], [66]. In *P. luminescens*, it is likely that the deficience of PhoP-dependent *pbgPE* expression is responsible for the avirulence of *phoP* mutant rather than the one of *ail1*_Pl_.

### What are the functions of Ail and PagC proteins in the life cycle of *Photorhabdus*?

To answer this question, we used a well-established assay by expressing in *E. coli ail* and *pagC* homologs from the *Photorhabdus* strains. Such strategy was successfully used for characterizing the role of proteins from the Ail/PagC/OmpX family in *Salmonella* and *Yersinia* in human serum resistance and invasion/adherence to eukaryotic culture cells [4]. Recombinant *Escherichia coli* clones, expressing cosmids from *Photorhabdus*, were also used in assays to study gain of toxicity against insects, nematodes, amoeba, and mammalian macrophages [67] and to attribute biological function to several *Photorhabdus* potential virulence loci [68]–[70]. With this heterologous assay, we showed that none of the over-expressed genes displayed a role in adherence to the tested mammal (CHO) or insect (Sl2b) cells. *Photorhabdus* life cycle is mainly extracellular except a transient invasive stage during the symbiont transmission to the new generation of IJ before they exit the insect cadaver [30]. This transmission stage is dependent on the production of the bacterial Mad pili [71]. By contrast with Ail and PagC from *Yersinia* and *Salmonella* species [5]–[8], our results do not suggest a role of Ail1, Ail2 or PagC in cell invasion. While *P. asymbiotica* ATCC43949 is a clinical isolate from human wounds resistant to human serum at 30°C and 37°C [37], it is surprising that neither *ail1*_Pa_ nor *pagC*_Pa_ participated in the human serum resistance. It is tempting to speculate that other *P. asymbiotica* proteins could be involved in serum resistance. Finally, when expressed in *E. coli*, only *ail2*_Pl_ and *pagC*_Pl_ appear to play a role in human serum resistance whereas *P. luminescens* TT01 was not described as a clinical isolate. One hypothesis could be that Ail2_Pl_ and PagC_Pl_ could play a role in resistance toward components of the insect blood, the hemolymph. Thus, we tested the resistance of recombinant *E. coli* bacteria toward different AMPs, a key component of the insect humoral insect immunity, though without any success. In pathogenic *Yersinia* species, Ail proteins bind substrates such as the host cell extracellular matrix proteins, fibronectin and laminin, as well as the complement regulatory proteins C4bp and factor H [72]. Therefore, in insects, Ail2_Pl_ and PagC_Pl_ may interact with similar hemolymph components yet to be identified.

### What is the evolutionary significance of the two Ail proteins in *P. luminescens* TT01, Ail1_Pl_ and Ail2_Pl_?

In *Y. pestis* KIM, four genes encoding the proteins from the Ail/PagC/OmpX family were identified including one OmpX protein (y1682) and three Ail proteins (y2446, y2034 and y1324). These three Ail proteins are phylogenetically distant and their corresponding genes are not adjacent on the *Y. pestis* KIM genome [73]. In *P. luminescens* TT01, we propose that the two *ail* genes result from a recent tandem duplication (see above). The genomic redundancy in prokaryotes can be explained as a consequence of three selective processes, (i) elevated protein dosage (identical and duplicated genes), (ii) protein diversification (divergent paralogs) and (iii) adaptation to environmental variations (ecoparalogs of intermediate divergence) [74]. The intermediate aminoacids identity between Ail1_Pl_ and Ail2_Pl_ suggests a case of ecoparalogs. Three clues also argue in favour of this hypothesis. First, as already described with ecoparalogs predicted to be on the outer membrane or in the periplasmic space where the environment influence is important for protein function and stability [74], Ail1_Pl_ and Ail2_Pl_ differ by their isoelectric point values (respectively, 9.0 and 7.0). Second, a usual role identified in the Ail/PagC/OmpX family, the resistance to human serum, was only conserved for one protein, Ail2_Pl_. Finally, regulation of the expression of *ail1*_Pl_ and *ail2*_Pl_ genes is obviously different and in the case of *ail1*_Pl_, we showed that the influence of external environment fluctuation by the way of magnesium concentration is important. All together, the existence of these two ecoparalogs is highly suggestive of an adaptation to multiple niches (at least, insect and nematode) in response to external fluctuation.

## Supporting Information

Figure S1 **Acetyl phosphate can phosphorylate PhoP-His** ***in vitro.*** To evaluate the efficiency of PhoP-His phosphorylation by acetyl phosphate, precast 12.5% polyacrylamide Mn^2+^-Phos-tag gel (Wako Chemicals, Japon) was used. When present the Phos-tag and its associated divalent cation Mn^2+^ form a complex with phosphorylated forms and retard protein migration. Four micrograms of purified PhoP-His were incubated *in vitro* either with 50 mM acetyl phosphate (lane 2) or without acetyl phosphate (lane 1) using the buffer described for EMSA protocol. SDS-PAGE was performed using standard protocols and gel was run at 4°C and 150 V to avoid phosphate hydrolysis until 10 min after loading blue sorting. Thereafter, the gel was incubated during 10 min in the Cathode buffer (40 mM 6-amino caproic acid, 25 mM Tris, 20% methanol) supplemented with 1 mM EDTA in order to quench Mn^2+^ cations and 20 min in the cathode buffer without EDTA to remove excess of EDTA. The gel was stained with coomassie brilliant blue. In absence of acetyl phosphate, only unphosphorylated PhoP-His is found (lane 1) whereas phosphorylated PhoP-His and dimerization are observed in presence of acetyl phosphate (lane 2) showing that acetyl phosphate can phosphorylate PhoP-His *in vitro*.
(JPG)

Table S1 **Primers used in this study.**
(DOCX)
