# Supplementary figures and images for: Ail and PagC-Related Proteins in the Entomopathogenic Bacteria of Photorhabdus Genus
Source: PLoS One. 2014 Oct 15;9(10):e110060. doi: 10.1371/journal.pone.0110060 (PMC4198210; doi:10.1371/journal.pone.0110060)

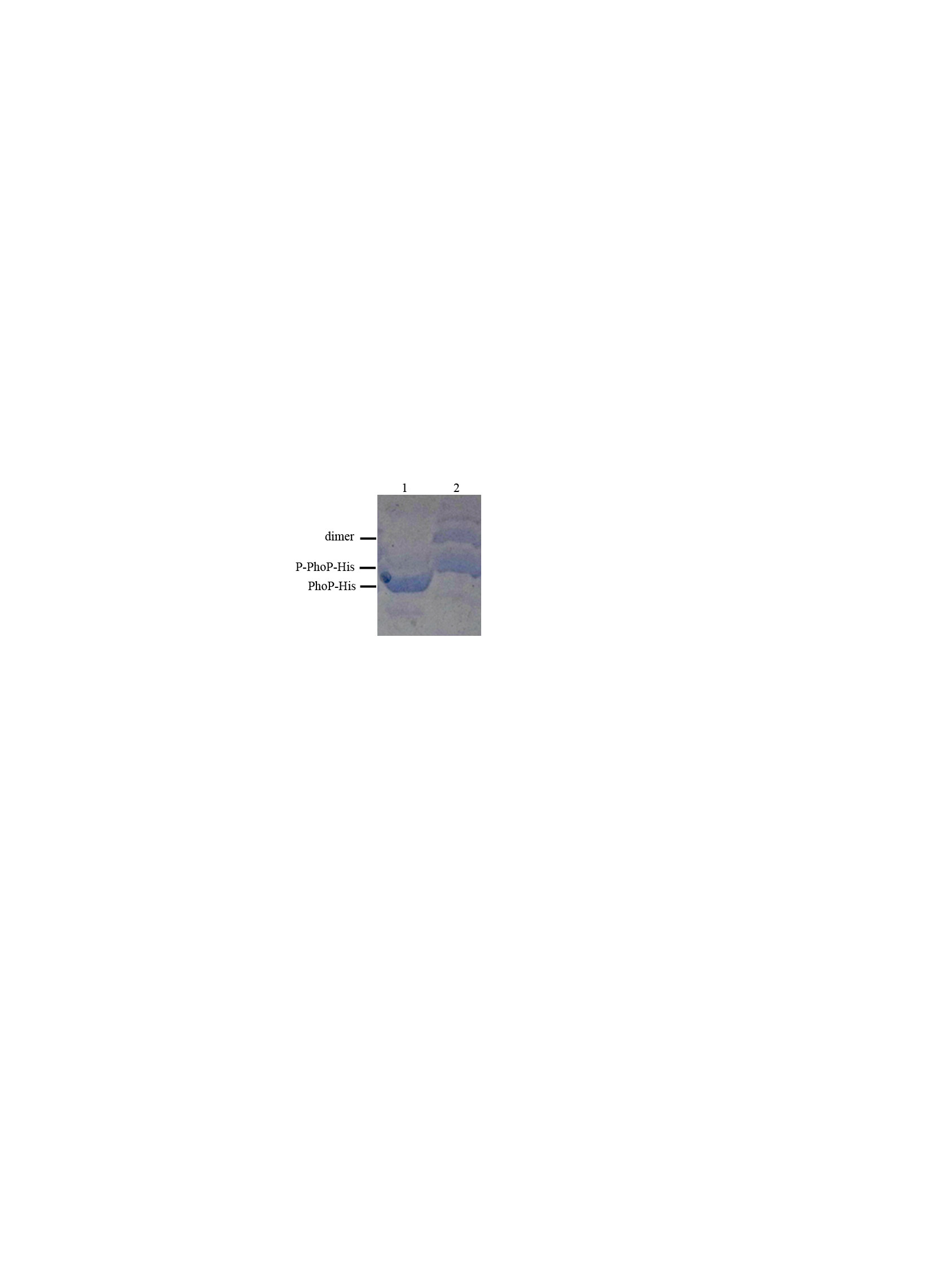

Supplement: Figure S1 — Acetyl phosphate can phosphorylate PhoP-His in vitro. To evaluate the efficiency of PhoP-His phosphorylation by acetyl phosphate, precast 12.5% polyacrylamide Mn2+-Phos-tag gel (Wako Chemicals, Japon) was used. When present the Phos-tag and its associated divalent cation Mn2+ form a complex with phosphorylated forms and retard protein migration. Four micrograms of purified PhoP-His were incubated in vitro either with 50 mM acetyl phosphate (lane 2) or without acetyl phosphate (lane 1) using the buffer described for EMSA protocol. SDS-PAGE was performed using standard protocols and gel was run at 4°C and 150 V to avoid phosphate hydrolysis until 10 min after loading blue sorting. Thereafter, the gel was incubated during 10 min in the Cathode buffer (40 mM 6-amino caproic acid, 25 mM Tris, 20% methanol) supplemented with 1 mM EDTA in order to quench Mn2+ cations and 20 min in the cathode buffer without EDTA to remove excess of EDTA. The gel was stained with coomassie brilliant blue. In absence of acetyl phosphate, only unphosphorylated PhoP-His is found (lane 1) whereas phosphorylated PhoP-His and dimerization are observed in presence of acetyl phosphate (lane 2) showing that acetyl phosphate can phosphorylate PhoP-His in vitro. (JPG) [file pone.0110060.s001.jpg]
