# Supplementary material for: Ail and PagC-Related Proteins in the Entomopathogenic Bacteria of Photorhabdus Genus
Source: PLoS One. 2014 Oct 15;9(10):e110060. doi: 10.1371/journal.pone.0110060 (PMC4198210; doi:10.1371/journal.pone.0110060)
Supplement: Table S1 — Primers used in this study. (DOCX) [file pone.0110060.s002.docx]

**Table S1:** Primers used in this study

| Primer name | Primer sequence (5’ to 3’) | Use |
| --- | --- | --- |
| y1324-*Pst*I | GCGCCTGCAGTTGGCTGGCCACTTTAGTCT | cloning of *ail* (y1324) from  *Yersinia pestis* CO92 |
| y1324-*Sac*I | GCGCGAGCTCTCAGCAATTTGAAACCACCA |  |
| plu2480-*Pst*I | GCGCCTGCAGCGCGCCTCGTAATTAATCTTT | cloning of *ail 1* (plu2480) from *Photorhabdus luminescens* TT01 |
| plu2480-*Sac*I | GCGCGAGCTCGATAACTGGGTGAGCACATCG |  |
| plu2481-*Pst*I | GCGCCTGCAGCCACGTTACATTTTGTTTTTCG | cloning of *ail 2*  (plu2481) from *Photorhabdus luminescens* TT01 |
| plu2481-*Sac*I | GCGCGAGCTCTGCACAGATCACAAAAGTTGG |  |
| Plu1967-*Pst*I | GCGCCTGCAGCAAATCGTTTAAATGCGTTAT | cloning of *pagC*  (plu1967) from *Photorhabdus luminescens* TT01 |
| Plu1967-*Sac*I | GCGCGAGCTCGACAAATCACCAAGGTTGTCA |  |
| PAU_02047-*Pst*I | GCGCCTGCAGCGCGATTGGCGATTAGTTAT | cloning of *ail*  (PAU_02047) from *Photorhabdus asymbiotica* ATCC43949 |
| PAU_02047-*Sac*I | GCGCGAGCTCCCTGTCACAAGACAGGGAATC |  |
| PAU_02601-*Pst*I | GCGCCTGCAGGTCGGTCTGCAAACCATTTT | cloning of *pagC* (PAU_02601) from *Photorhabdus asymbiotica* ATCC43949 |
| PAU_02601-*Sac*I | GCGCGAGCTCAATCACCAAGGCGATAAATCA |  |
| L-plu2480Eco | CGGAATTCAAAGCGGGTATCCAGGTTTA | cloning of the region upstream *ail 1* from *Photorhabdus luminescens* TT01 |
| R-plu2480Bam | CGGGATCCCCTACCGCTACCACTGAAGC |  |
| L-plu2481Eco | CGGAATTCCTGAATGGTACTCTTAATTACGC | cloning of the region upstream *ail 2* from *Photorhabdus luminescens* TT01 |
| R-plu2481Bam | CGGGATCCCTGCCACTGCTGAGGCTAT |  |
| L-gyrB | ATACACGAAGAAGAAGGTGTTTCAG | qRT-PCR of an internal region within *gyrB* from *Photorhabdus luminescens* TT01 |
| R-gyrB | TACCTGTCTGTTCAGTTTCTCCAAC |  |
| L-plu2480 | agaacattagtggcttcagtggtag | qRT-PCR of an internal region within *ail 1* from *Photorhabdus luminescens* TT01 |
| R-plu2480 | attatccaactcgtagcggtatttc |  |
| L-plu2481 | AGCAAGATTGGTACTGGTGATTTAG | qRT-PCR of an internal region within *ail 2* from *Photorhabdus luminescens* TT01 |
| R-plu2481 | CATAAGCAGCAGAGGTCTTACTCTC |  |
| L-plu1967 | GTATCTGCGATAACTTTACCTGCTC | qRT-PCR of an internal region within *pagC* from *Photorhabdus luminescens* TT01 |
| R-plu1967 | CATTATAGCTGTAGCCAAGATGGAC |  |
| L- PhoP-His-*Nde*I | CGCGCGCCGCATATGCGGATATTGATCGTTGAAGACAACATGTTACTGC | Cloning of *phoP* from *P. luminescens* TT01 |
| R- PhoP-His-*Bam*HI | GCGCGGATCCTTACACATCGAAGCGATAGCCTTGGCCCCG | Cloning of *phoP* from *P. luminescens* TT01 |
